# Vim thalamotomy in a patient with Holmes’ tremor and palatal tremor - Pathophysiological considerations 

**DOI:** 10.1186/s12883-015-0277-5

**Published:** 2015-03-11

**Authors:** Futaba Maki, Sumito Sato, Katsushige Watanabe, Toshiyuki Yanagisawa, Yuta Hagiwara, Takahiro Shimizu, Yasuhiro Hasegawa

**Affiliations:** Department of Neurology, St. Marianna University School of Medicine, Kawasaki, Kanagawa 216-8511 Japan; Department of Neurosurgery, Kitasato University School of Medicine, Kanagawa, Japan; Department of Neurosurgery, Tokyo Metropolitan Matsuzawa hospital, Tokyo, Japan

**Keywords:** Holmes’ tremor, Palatal tremor, Ventral intermediate nucleus (Vim) thalamotomy

## Abstract

**Background:**

We peformed a ventral intermediate nucleus (Vim) thalamotomy in a patient with Holmes’ tremor and palatal tremor. The frequencies of these movement disorders were 4 Hz and 3 Hz, respectively. Vim thalamotomy stopped the Holmes’ tremor but not the palatal tremor. Our observations suggest different mechanisms for these two involuntary movements.

**Case presentation:**

A 57-arm 11 months after a pontine hemorrhage. Transoral carotid ultrasonography revealed periodic motion of her posterior pharyngeal wall with a frequency of 3 Hz. Recording of neuronal activities in the thalamus revealed a 4Hz rhythmic discharge time that was associated with her tremor in the contralateral arm. A left Vim thalamotomy was performed. The resting tremor of the upper limb stopped, but the kinetic tremor recurred 6 months after the thalamotomy. No effect was observed on her palatal tremor.

**Conclusions:**

The different effects of Vim thalamotomy on the Holmes’ tremor and palatal tremor suggest different oscillation sources for these two involuntary movements.

## Background

Holmes’ tremor is defined by the Ad Hoc Scientific Committee of the Movement Disorder Society as a general term for symptomatic tremors, including midbrain tremors, rubral tremors, thalamic tremors, and tremors in Benedict’s syndrome [1]. Characteristics of Holmes’ tremors include that they <1 > have a delayed onset from 4 weeks to 2 years after a stroke; <2 > are low-frequency (≤4.5 Hz) tremors primarily of proximal muscles; and <3 > are rest, action, and postural tremors that increase with movement. Reported foci include the area superolateral to the red nuclei, rubrothalamic tract, central tegmental tract, superior cerebellar peduncle, and the substantia nigra [2]. Drug therapy including L-dopa, anticholinergic agents, and zonisamide is effective in some cases [3]. This suggests dysfunction of the nigrostriatal tract [4], which is thought to be involved in the onset of rest tremors.

Moreover, in patients with midbrain tremors, 2β-Carbomethoxy-3β-(4-(123) I-iodophenyl) tropane ((123) I-beta-CIT) SPECT shows decreased uptake in the putamen and caudate nucleus on the side of the lesion, thus suggesting a relationship between these sites and symptoms [5]. These same foci are also involved in palatal tremor, but the pathogenesis varies among patients. Many cases are resistant to oral drug therapy, and the pathophysiology is unclear. We recently performed a ventral intermediate nucleus (Vim) thalamotomy in a patient with Holmes’ tremor and palatal tremor, and neuronal activity in the thalamus was observed. We now discuss the pathophysiology in this case based on characteristic findings.

## Case presentation

A 57-year-old right-handed woman with hypertension suddenly developed right hemiparesis and ataxia in 2010. Eleven months after the left pontine tegmentum hemorrhage, she developed a resting tremor in her right upper extremity. Trihexyphenidyl HCl, levodopa/carbidopa hydrate, and arotinolol were prescribed for outpatient use but were not clearly effective. Thus, 2 years after the cerebral hemorrhage, she was admitted to the hospital for surgical treatment. On admission, no abnormal findings were seen with a general physical examination. The patient was lucid but had ataxic dysarthria and palatal tremor. She also had mild right hemiplegia and a cerebellar ataxic gait. A coarse tremor was present, primarily of the proximal right upper extremity at rest and with posture that increased with movement. The deep tendon reflexes were normal, but superficial sensation in the right upper extremity was decreased.

**Figure 1:**
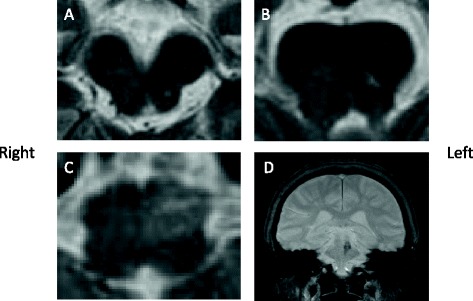
**Magnetic resonance images 11 months after the onset of hemorrhage.** The hemorrhagic focus in the left tegmentum (**A**, **B**, T2-weighted images). High signal intensity in the left medulla oblongata indicating hypertrophic olivary degeneration (**C**, T2-weighted image). Hemosiderin ring around the lesion in the left pontine tegmentum (**D**, coronal view, Fast Field Echo-weighted image).

**Figure 2:**
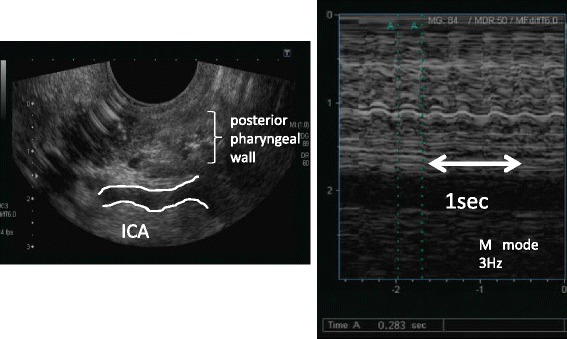
**Measurement of frequency of the oral myoclonus with transoral carotid ultrasonography.**

Hematologic and biochemical blood tests were normal. Magnetic resonance imaging (MRI) showed an old focus of the hemorrhage in the left pontine tegmentum, and T2-weighted imaging revealed a high signal intensity area in the left inferior olivary nucleus of the medulla oblongata (Figure 1). A surface electromyogram showed irregular grouped discharges at a rate of about 4 Hz in the brachialis and forearm muscles. Transoral carotid ultrasonography [6] was performed to evaluate the palatal abnormal movement. M mode imaging revealed rhythmic movements of the levator veli palatini muscle and pharyngeal wall with a frequency of 3 Hz; this was diagnosed as palatal tremor (Figure 2). Subtotal scores of Part A (tremor localization/severity rating), Part B (specific motor task/ function rating) and Part C (functional disabilities resulting from tremor) of the Clinical Rating Scale for Tremor (CRST) [7] were 10, 19, and 20, respectively, and a total score of CRST was 39.

**Figure 3:**
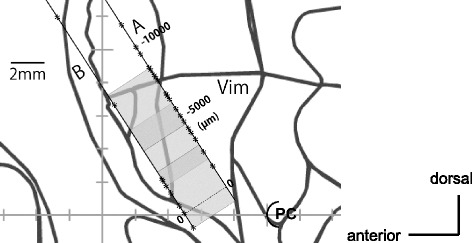
**Sagittal plane of the left thalamus 15.5 mm lateral from the midline.** The recording trajectories (**A** and **B**, 3 mm apart) and the points (asterisks) where 4-Hz rhythmic discharges were found along the trajectories. Tentative target, 0 (zero point), on the intercommissural line. Shaded rectangular areas depict coagulated lesions made with three applications of dual coagulation needles. PC: posterior commissure.

The patient did not want deep brain stimulation (DBS) using an implantable device, so we performed a left Vim thalamotomy. The patient’s head was fixed in a Leksell stereotactic frame, and based on MRI, the tentative target was set on a line connecting the anterior commissure and posterior commissure, 5.5 mm anterior to the posterior commissure, and 15.5 mm left lateral from the midline. Under local anesthesia and using semi-microelectrodes, simultaneous recordings were obtained on a track toward the tentative target (Track B) and on another track that was parallel and 3 mm posterior (Track A) (Figure 3).

**Figure 4:**
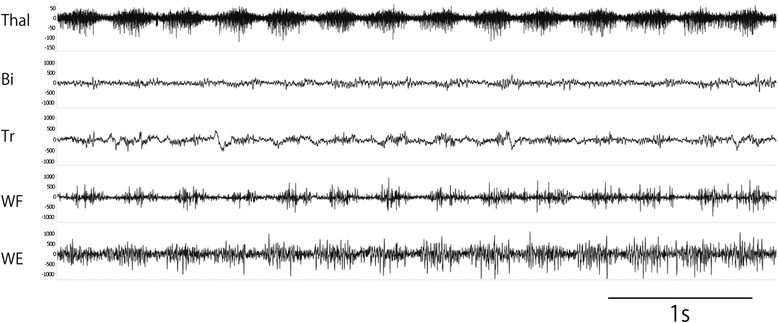
**Rhythmic grouped discharges recorded 3410 μm from zero in the thalamic Vim.** These discharges were time locked with the contralateral peripheral tremor. The top trace was recorded from the left thalamic Vim nucleus, and electromyographic recordings (from the second to the fifth traces) were from the right upper extremity. B, biceps muscle; T, triceps muscle; WF, wrist flexor muscle; WE, wrist extensor muscle.

On Track A, at 10,410 μm dorsal around the Vim nucleus, approximately 4Hz rhythmic discharges corresponding to the tremor rhythm were intermittently observed in a wide area (Figure 4). These were observed near the tentative target on Track B. Even when the tremors were passively stopped, the rhythmic discharges continued. Almost no kinesthetic response to passive motion of the limbs could be recorded, and the somatotopic organization of the Vim nucleus was lost. In addition, when the sites where these rhythmic discharges were recorded were widely coagulated, the resting tremors almost immediately disappeared. The palatal tremor was unchanged compared to before surgery. After the surgery, a total score of CRST was decreased from 39 to 20. Scores of Part A, Part B and Part C were 3, 5, 12, respectively.

The resting tremor stopped during the month after surgery, almost no action tremor was observed, and the patient was able to practice handwriting. Subsequently, the action tremor again gradually appeared. On evaluation after 6 months, no resting tremor was seen, but about a 2.5Hz (slower than before surgery) coarse tremor with posture and movement was present. The palatal tremor remained unchanged compared to before surgery. The CRST score increased from 20 to 42, and subtotal scores of Part A, B and C were 7, 15, and 20, respectively.

## Discussion

Damage to the cerebellothalamic tract is thought to be involved in Holmes’ action tremors [1].Our patient also had disruption of afferent pathways (deafferentation) from the cerebellar dentate nucleus to the thalamus due to a brainstem lesion, which presumably led to thalamic functional changes. In other words, this suggests that the approximately 4-Hz rhythmic discharges observed in the thalamus during surgery that corresponded to the tremor rhythm contributed to the development of the tremor.

Meanwhile, palatal tremor is due to disruption of pathways from the dentate nucleus to the inferior olivary nucleus via the central tegmental tract, and is caused by intrinsic spontaneous firing of cells in the inferior olivary nucleus [8]. Inhibitory fibers from the contralateral cerebellum are usually distributed to gap junctions between inferior olivary nucleus cells, and this inhibits electrotonic coupling between cells [8]. When lesions occur as in our patient, the cerebellar inhibitory tracts are disrupted, inhibition of electrotonic coupling is lost, and synchronous rhythmic discharges occur [8,9]. A similar oscillatory mechanism, as in the inferior olivary nucleus, is also thought to exist in the thalamus [10].

**Figure 5:**
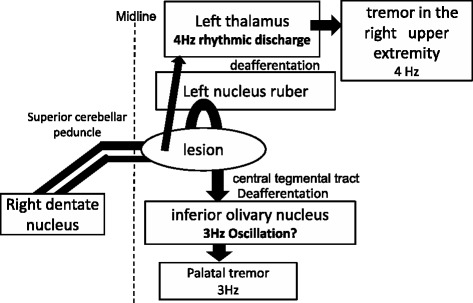
**Proposed mechanism of why even though the upper extremity tremor resolved with Vim thalamotomy,** **the palatal tremor was unaffected.**

MRI in our patient showed findings suspicious of pseudohypertrophy of the inferior olivary nucleus, thus suggesting disruption of the dentate-olivary tract. This resulted in about 3-Hz oscillations in the inferior olivary nucleus, thus leading to palatal tremor. Because the origins of the upper extremity tremor and palatal tremor were mutually independent, their frequencies of 4 Hz and 3 Hz were slightly different. This is probably why even though the upper extremity tremor resolved following Vim thalamotomy, the palatal tremor was unaffected (Figure 5). Our patient had approximately 4-Hz rhythmic discharges in a wide area around the Vim nucleus, and somatotopic organization was lost. These types of marked functional changes in the thalamus (excessive synchronous activity and loss of somatotopic organization) can make treatment difficult. Interestingly, at 6 months after surgery, the resting tremor was still suppressed, but the postural and action tremors had recurred, and their rhythm was slower than before surgery. Although details about the mechanism are unclear, Vim thalamotomy reduced the tremor component, but the original ataxic component may also have become more prominent.

Surgical treatment for Holmes’ tremors includes thalamotomy targeting the thalamic Vim [11,12], DBS [13-16], and a combination of subthalamic nucleus DBS [17]. However, these treatments are often only partially effective, including cases with improvement in only distal and action tremors, and other cases with a short duration of effectiveness. For proximal tremors in which control with Vim-DBS has been difficult, coagulation of the internal segment of the globus pallidus is reported to be effective [18,19]. Globus pallidus output may also be involved in Holmes’ tremors, and since 2000, the effectiveness of zona incerta DBS has been gaining attention [20].Resolution of palatal tremor, however, has not been described in these reports [12,18].

## Conclusion

We performed a Vim thalamotomy in a patient with Holmes’ tremor and palatal tremor after a brainstem hemorrhage. Using semi-microelectrode recordings, rhythmic discharges corresponding to the tremor rhythm were observed in a wide area around the Vim nucleus of the thalamus, suggesting involvement of this site in the upper extremity tremors. The tremors temporarily resolved after coagulation of these thalamic neurons, but they recurred in a few months, and thus, the improvement was limited. These types of functional changes in thalamic neurons over a wide area, cerebellar ataxia, and the existence of multiple independent sites of origin can make treatment difficult.

### Consent

Written informed consent was obtained from the patient for the publication of this case report and accompanying images.
